# Two years of COVID-19: persistently reduced well-being and increases in global psychopathology during the pandemic in a representative Austrian population-sample within the COH-FIT study

**DOI:** 10.3389/fpsyt.2026.1783600

**Published:** 2026-06-01

**Authors:** Monika Schlögelhofer, Elena Aschauer, Harald Aschauer, Christoph U. Correll, Georg Dorffner, Alexa Kuenburg, Marco Solmi, Trevor Thompson

**Affiliations:** 1 Biopsychosocial Corporation, BioPsyC, Non-profit Association for Research Funding Ltd., Vienna, Austria; 2 Abteilung für Psychiatrie und Psychotherapeutische Medizin mit Zentrum für Psychosomatik, Klinik Hietzing, Wiener Gesundheitsverbund, Vienna, Austria; 3 Department of Child and Adolescent Psychiatry, Charité Universitätsmedizin Berlin, Berlin, Germany; 4 The Zucker Hillside Hospital, Northwell Health, New York, NY, United States; 5 Donald and Barbara Zucker School of Medicine at Hofstra/Northwell, New York, NY, United States; 6 Deutsches Zentrum für Psychische Gesundheit (DZPG), Partner Site Berlin, Berlin, Germany; 7 Center of Medical Data Science, Institute for Artificial Intelligence, Medical University of Vienna, Vienna, Austria; 8 Klinik für Konsilarpsychiatrie und Psychosomatik, Universitätsspital Zürich, Zürich, Switzerland; 9 Department of Psychiatry, University of Ottawa, Ottawa, ON, Canada; 10 Department of Mental Health, The Ottawa Hospital, Ottawa, ON, Canada; 11 Ottawa Hospital Research Institute, Ottawa, ON, Canada; 12 Early Psychosis Interventions and Clinical-Detection Lab, Department of Psychosis Studies, Institute of Psychiatry, Psychology & Neuroscience, King’s College, London, United Kingdom; 13 Department of Psychology, Faculty of Education, Health and Human Sciences, University of Greenwich, London, United Kingdom

**Keywords:** Austria, COH-FIT, coping strategies, COVID-19 pandemic, mental health, pandemic related stressors, psychopathology (‘P-score’), repeated cross-sectional representative study

## Abstract

**Introduction:**

The COVID-19 pandemic worsened well-being and mental health worldwide, but effects have diminished over time. However, prospective national data within representative samples remain scarce. We aimed to examine the change in well-being and psychopathology from pre-pandemic to intra-pandemic times in an Austrian representative general population sample, to identify vulnerable subgroups, and explore most effective coping strategies to mitigate the impact of COVID-19.

**Methods:**

Data were collected in Austria as part of the Collaborative Outcomes Study on Health and Functioning During Infection Times (COH-FIT) survey, an international, multilingual, anonymous online survey assessing mental health indicators during COVID-19. Adults ≥18 years old participated through nationally representative sampling across three waves from 05/2020-04/2022. Outcomes included the WHO well-being index (WHO-5) and a global psychopathology score (‘P-score’), alongside 12 predefined risk factors and 16 coping strategies.

**Results:**

Across 4,148 adults, the mean WHO-5 well-being score decreased by 7.5 ± 17.7 points from the pre-pandemic baseline (73.2 ± 19.7) to the intra-pandemic average (65.7 ± 24.1) (p<.001). Participants with female sex, pre-existing mental or physical health conditions, and unemployment experienced greater declines. The proportion of individuals scoring <50, indicating depression, increased from 12.6% pre-pandemic baseline to 25.1% intra-pandemic, and the proportion scoring <29, indicating major depression, increased from 3.3% to 9.7% (both p<.001). The ‘P-score’ increased by 9.6 ± 15.0 points from 24.1 ± 19.5 pre-pandemic baseline to 33.7 ± 22.4 intra-pandemic (p<.001) with the same risk groups (except female sex). Although the greatest deterioration in both outcomes occurred during the mid-pandemic period (04/2021), neither well-being nor ‘P-score’ levels returned to pre-pandemic baseline values by 04/2022, nor to values from 05/2020 (Wave 1). Greater deterioration in WHO-5 and the P-score were associated with female sex, unemployment, pre-existing mental or physical disorders, and COVID-19 infection. The most commonly reported helpful coping strategies included internet use, physical activity, media consumption, social media and remote interaction, and meaningful hobbies.

**Discussion:**

COVID-19 had a persistent negative impact on well-being and mental health in Austria. Vulnerable subgroups - including those with prior health conditions and unemployment - were particularly affected. The findings underscore the importance of implementing public health measures together with targeted interventions, preventive measures, and long-term psychosocial support, especially for risk populations.

## 1 Introduction

The global outbreak of the COVID-19 pandemic, caused by the novel coronavirus SARS-CoV-2 in March 2020, has profoundly altered everyday life across the world. As of March 2026, over 779 million individuals have been infected, and more than 7.1 million deaths have been reported globally (World Health Organization WHO Dashboard; https://covid19.who.int). Although the COVID-19 pandemic has officially been declared over, the risk of future outbreaks remains considerable, underscoring the importance of investigating individuals´ responses to pandemic-related stressors. Furthermore, Long COVID represents a substantial public health concern, with an estimated 36 million cases reported in the WHO European Region. This condition frequently manifests with neuropsychological symptoms and is often associated with myalgic encephalomyelitis/chronic fatigue syndrome (ME/CFS) (1).

The existing literature on the psychological impact of the COVID-19 pandemic is heterogeneous, with studies reporting varying sizes and durations of effect. Early meta-analytic evidence, including a systematic review of 25 studies involving over 72,000 participants (2) suggested that lockdowns had only small adverse effects on mental health, with most individuals demonstrating psychological resilience and modest, short-lived consequences (2). Similarly, a meta-analysis of 65 studies from 2020 found a small initial increase in mental health symptoms in early 2020, which largely disappeared by mid-2020, though symptoms of depression remained somewhat elevated (3). A further meta-analysis of 41 studies until August 2021 found higher rates of psychological distress in certain subgroups, but substantial heterogeneity between studies made it difficult to draw general conclusions about long term effects (4).

Other studies, however, point to more persistent and wide-ranging effects. Dragioti et al. (5) included 173 peer-reviewed observational studies until September 2020, reported increased mental health problems – particularly anxiety, depression, stress, and sleep disturbances – especially among females, individuals infected with COVID-19, and residents of low- and middle-income countries. This pattern was also observed at the national level: a repeated cross-sectional study in Sweden, for instance, found significant increases in anxiety and depression alongside declines in self-rated health and quality of life, with higher income and education appearing to soften these effects (6). Importantly, the magnitude of these effects varied considerably across countries, as demonstrated by a cross-sectional study across 14 Western European nations, which found substantial variation in well-being, with Austria performing comparatively better than most other nations (7). Notably, however, a multinational analysis using only representative samples from eight European countries found no overall change in depression and anxiety rates between pre-pandemic and pandemic periods, though stricter public health measures were still linked to small increase in emotional distress (8).

Taken together, while the evidence is mixed, the broader literature suggests that certain groups (including individuals with pre-existing mental health or physical health conditions, women, and those facing socioeconomic disadvantage) may be at greater risk for pandemic-related psychological difficulties (4, 5). Among the coping strategies investigated, exercise, social contact, and internet use were reported as the most effective in one of the largest international pandemic surveys to date (9). However, evidence on coping remains limited and mixed, with one study across Austria and Italy finding that maladaptive coping strategies were consistently linked to greater psychological distress, while adaptive strategies were associated with reduced stress (10).

In Austria, the COVID-19 pandemic was met with particularly early and strict public health measures, including lockdowns, curfews, and bans on public gatherings. A cross-sectional survey conducted in spring 2022 found that rates of depression had increased compared to April 2020, with strongest effects seen in young people, females, and those with lower income, while anxiety, insomnia, and stress levels did not change significantly (11). The Austrian Corona Panel Project, a large repeated- measures study with 30 waves of data collection between 2020 and March 2022, found that subjective well-being did not show large overall changes, but varied more with the strictness of lockdown measures than with COVID-19 infection rates (12). While these studies offer valuable insights, the existing Austrian evidence has important limitations: studies have largely focused on specific pandemic phases or situations such as lockdowns (13–15), used non-representative samples, or measured only a limited range of mental health outcomes. Importantly, no study so far has looked at trajectories of both well-being and global psychopathology across the full pandemic period using a population-representative repeated cross-sectional design in Austria.

The present study was designed to fill this gap. The current study aimed to evaluate the trajectories of well-being (WHO-5, range 0 - 100) and global psychopathology (validated composite ‘P-score’, range 0 - 100) in the Austrian general population across three time points during the COVID-19 pandemic, using a repeated cross-sectional population-representative survey design. Based on the existing literature, we expected well-being would decline and psychopathology would increase over the course of pandemic at least during certain pandemic phases and particularly among vulnerable subgroups. In addition, the identification of effective coping strategies was included as an exploratory aim, given the limited and mixed evidence available in this area. As the acute phase of the COVID-19 pandemic has passed, these findings may help identify vulnerable subgroups and inform evidence-based public health strategies aimed at strengthening mental health preparedness for future public-health crises in Austria.

## 2 Materials and methods

### 2.1 Study design

The present study reports data of a representative sample collected in three repeated cross-sectional surveys (waves) in adults in Austria over a two-year period during the COVID-19 pandemic as part of the global Collaborative Outcomes Study on Health and Functioning (COH-FIT) survey, a cross-sectional, multi-language, online, anonymous, survey in the general population (children, adolescents, and adults) on an individual basis with a longitudinal design on a population basis (9, 16–19). The COH-FIT survey (both, snow-ball non-representative and representative sampling) started on April 27, 2020, and collected demographic, environmental, psychosocial and psychological variables and investigated, among others, physical and mental health, social and behavioral outcomes during the pandemic. The survey was designed by the international COH-FIT team and was provided on the COH-FIT website for online participation (www.coh-fit.com). The representative data collection was based on the Computer-Assisted Web Interviewing (CAWI) technique using different polling agencies. Details of COH-FIT methods e.g., survey translations, survey platform, data management and protection, study registration statement are described elsewhere (16, 17).

The COH-FIT surveys in the Austrian web-active representative adult population were administered by the “Das Österreichische Gallup Institut” (www.gallup.at) via CAWI (Computer-Assisted Web Interviewing) technique. The sample from the online access panel was stratified using quota sampling based on gender, age, and federal state of residence. Education and employment status were additionally monitored as “soft quotas” during recruitment but were not strictly enforced, which may explain remaining deviations from population statistics. Post-stratification weighting (raking/iterative proportional fitting) was subsequently applied to further align the sample with population distributions for the quota variables. We compared the percentage of respondents in specific categories (gender, age groups, employment, education) in the sampled data to population statistics (see **Supplementary Table 1**). The online access panel used for this COH-FIT survey adheres to stringent quality criteria, including measures to prevent false or multiple registrations through methods such as manual screening and automated verification algorithms. Additionally, participant authentication is based on bank details, and a double opt-in procedure is in place. The panel maintains comprehensive profile data, which allows for precise participant selection. Furthermore, the company strictly adheres to regulations designed to protect the data and privacy of panelists.

### 2.2 Ethics committee

The global survey was launched on the COH-FIT website after the first ethics committee approval by Aristotle University of Thessaloniki, Greece. In Austria, the project was submitted and approved by the ethics committee of the Medical University of Vienna. The committee stated and determined that participation in the survey was voluntary, the participation procedure was anonymous, and therefore the survey did not require the request for written informed consent. The study was performed in accordance with the ethical standards laid down in the 1964 Declaration of Helsinki and its later amendments. The manuscript does not contain clinical study or individual patient data.

### 2.3 Recruitment, participants

**Figure 1:**
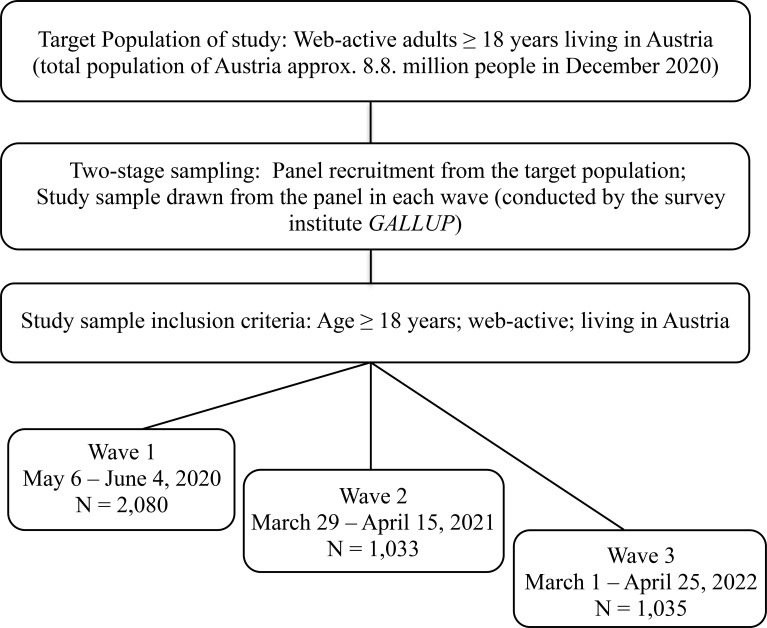
Flow diagram of the study sample.

According to official statistics, the adult population (≥18 years) of Austria was approximately 8.8 million in December 2020. Although the selection of one specific year within the 2-year period is inherently arbitrary, a decision was required. Therefore, population statistics from 2020 were used, as they were not yet affected by potentially biased mortality rates related to the pandemic. The survey targeted representative web-active adults aged 18 years and older living in Austria. Every person in the web-active adult population had the same chance of becoming part of the sample. The sampling was two-stage: in the first step, panel participants were recruited from the population, in the second step, the sample was drawn from the panel. In Austria, three waves of representative data collection took place: Wave 1: May 6^th^ to June 4^th^ 2020 (N = 2,080), Wave 2: March 29^th^ to April 15^th^ 2021 (N = 1,033) and Wave 3: March 1^st^ to April 25^th^ 2022 (N = 1,035). Participants were not followed longitudinally. Each wave represents an independent sample. Participants provided both current and retrospective assessments covering the pre-pandemic and intra-pandemic periods. A study flow diagram illustrating participant inclusion across the three survey waves is provided in **Figure 1**.

### 2.4 Outcomes

There were two co-primary outcomes: changes in well-being and changes in psychopathological symptoms (‘P-score’). Well-being was measured with the World Health Organization-Five Well-Being Index (WHO-5 well-being index) (20), a short rating scale consisting of five items measuring subjective well-being. Answer options were converted from the six-points Likert items to Visual Analogue Scale (VAS) ratings from 0 - 100. The WHO-5 well-being index has adequate validity and has been applied successfully across a wide range of studies. The score is calculated by adding up the score of each of the five items. It gives a final score from 0 (worst) to 100 (best well-being). In general population studies, the mean WHO-5 well-being score is 70 (21). In addition to the raw continuous scores, we also computed binary WHO-5 well-being scores based on two clinical thresholds. First, we used a cut-off of <50, as this is used as an indicator for testing for depression (20) and e.g., has been associated with significantly higher mortality rates in patients with cardiac disease (22). Second, we used a threshold of <29, as this has been suggested to be indicative of clinical, major depression (20).

‘P-score’ is a composite psychopathology measure. Methods, rationale for the scoring method as well as the results of the development and validation of the ‘P-score’ are described in detail elsewhere (18). The P-score is composed of five domains: anxiety, depression, post-traumatic stress disorder, psychosis, and psychophysiology (stress, sleep, concentration). The ‘P-score’ assessment underwent an internal validation procedure (18) and concurrent validation by computing correlations with validated full-length measures for the same constructs and domains during the current COH-FIT survey which are detailed in the COH-FIT-Adult design publication (16). Only domains with at least moderate correlations (r ≥ 0.50) with their respective validated full-length questionnaires were considered as acceptable to be included as a component of the composite ‘P-score’ (18). A total of 13 items were used for the five domains for the ‘P-score’. The mean item score for each of the five domains was computed and then averaged to create an overall ‘P-score’. Analysis of a previous sample of over 100,000 survey respondents supported the five-dimensional domain structure and the existence of a higher order general P-score factor, and found good reliability for both the five domain-specific factors (ω=.71-.91) and the composite P-score factor (ω=.95) (18). The ‘P-score’ thus represents a multidimensional measure of symptoms of different psychopathologic domains ranging from 0 (best) to 100 (worst psychopathology) (18).

Changes in the WHO-5 well-being index and ‘P-score’ were measured within the last two weeks before the beginning of the pandemic (quote: “2 weeks of regular life prior to the outbreak of the COVID-19 pandemic”) and the last two weeks before the specific wave of collection (quote: “last two weeks prior to taking the survey”).

### 2.5 At-risk groups and coping strategies

Based on previous literature during the COVID-19 pandemic (5, 23), we *a priori* identified 11 risk factors for poor well-being and mental health (‘P-score’), namely having had a COVID-19 infection, being under age of 30 years old, being female, being unemployed, having healthcare worker employment, having had a mental disorder, having had a physical disorder, having first-generation immigrant status, living in a large city, being obese (calculating body mass index (BMI) 30 or higher based on self-reported height and weight), and having lost someone due to COVID-19 (9).

Additionally, respondents were invited in each survey wave to rate the importance (“very important”, “somewhat important”, “not important”) of the following previously defined sixteen coping strategies for effectively dealing with and managing the pandemic and its effects. The coping strategies were: internet use, exercise and walking, media use (TV, movies, radio, music), social media use and remote social interactions, meaningful hobby, keeping informed about the COVID-19 pandemic, direct personal contact and social contact or interactions, spending time with a pet, physical intimacy and sexual activity, prescribed medications, work (on site or from home), gaming, studying or learning something new, religion and meditation and spirituality, use of substances (tobacco, alcohol, other), and “other” strategies (9).

### 2.6 Statistical analysis plan

Overall change in WHO-5 well-being and ‘P-scores’ from before to during the pandemic were examined with paired t-tests, and the change in proportion of WHO-5 well-being McNemar’s χ^2^ test. The magnitude of change in WHO-5 well-being scores and ‘P-scores’ across the pandemic were also compared for subjects with versus without each risk factor using independent t-tests.

For missing values data were imputed using predictive mean matching for continuous values and logistic regression for categorical variables (24).

We also explored a potential recall bias for WHO-5 well-being index and ‘P-score’, conducting polynomial regression analyses with linear or quadratic relationships. We also compared whether overall change from WHO-5 well-being and ‘P-score’ pre-pandemic baseline to during-pandemic (intra-pandemic) scores differed across the three survey wave dates, using between-groups ANOVA with polynomial contrasts. To examine whether respondents retrospective recall of their pre-pandemic baseline well-being and ‘P-scores’ was influenced by the date on which the survey was completed, ANOVA was performed. Survey wave was as an independent variable and WHO-5 Well-Being Index and ‘P-score’ pre-pandemic baseline scores were the dependent variable.

To corroborate the five-domain ‘P-score’ structure (anxiety, depression, post-traumatic stress disorder, psychosis, and psychophysiology) in this Austrian sample, we performed confirmatory factor analysis using a correlated 5-factor model. The following model fit criteria were used to indicate adequate model fit: Comparative Fit Index (CFI) >0.95, Root-Mean-Square Error of Approximation (RMSEA) <.06, and Standardized Root-Mean-Square Residual (SRMR) <0.08 (25). Mean-adjusted maximum likelihood was used to estimate parameters with robust standard errors and Satorra-Bentler Scaled Test Statistics to account for any potential non-normality (Satorra & Bentler, 2010). Overall, ‘P-score’ and individual domain reliabilities were estimated with coefficient omega (25).

All analyses were performed in R (R Foundation for Statistical Computing, 2019), were two-sided and with an adjusted alpha criterion of α=.001 to provide a more stringent control for multiplicity of testing.

## 3 Results

### 3.1 Survey sample

Overall, a total of 4,148 adults (age = 47.0 ± 16.0 years (range = 18–91); 47.3% men, 52.5% women, 0.07% non-binary, 0.07% transgender or intersexual) provided data across three representatives survey waves. The characteristic descriptive data of survey respondents, numbers, and percentages of groups across Waves 1–3 and specific questions of the survey are shown in **Table 1**. The study sample and 2020 general Austrian population statistics were generally well-aligned with small differences for sex (1.4%), and age group (0.8-2.3%), and somewhat larger differences for education level (0.1-10.6%). Detailed comparisons are provided in **Supplementary Table 1**.

**Table 1:** Participant characteristics and numbers and percentages by survey wave 1–3 and respective questions asked during survey.

Characteristics and respective questions	Group	Wave 1 (May 2020)		Wave 2 (April 2021)		Wave 3 (April 2022)	
		N	%	N	%	N	%
Sample Size		2,080		1,033		1,035	
Age ^*^	18-34	526	25.3%	247	23.9%	271	26.2%
	35-49	538	25.9%	258	25%	252	24.3%
	50-64	673	32.4%	360	34.8%	301	29.1%
	65+	343	16.5%	168	16.3%	211	20.4%
Gender ^2*^	Male	967	46.5%	491	47.5%	506	48.9%
	Female	1,110	53.4%	541	52.4%	527	50.9%
	Non-binary	2	0.1%	1	0.1%	0	0%
	Transgender or intersex	1	0%	0	0%	2	0.2%
Ethnicity ^3*^	White	2,007	96.5%	997	96.5%	1,002	96.8%
	African/African descent	6	0.3%	1	0.1%	3	0.3%
	Hispanic	5	0.2%	3	0.3%	2	0.2%
	Asian	18	0.9%	11	1.1%	5	0.5%
	Mixed	35	1.7%	11	1.1%	11	1.1%
	Other	9	0.4%	10	1%	12	1.2%
Education ^4*^	None	6	0.3%	1	0.1%	2	0.2%
	Primary school	16	0.8%	12	1.2%	12	1.2%
	High school	1,480	71.2%	749	72.5%	802	77.5%
	College/university degree	553	26.6%	255	24.7%	214	20.7%
	PhD	25	1.2%	16	1.5%	5	0.5%
Socio-economic-status ^5*^	0-24	58	2.8%	26	2.5%	43	4.2%
	25-49	331	15.9%	159	15.4%	95	9.2%
	50-74	1,342	64.5%	653	63.2%	758	73.2%
	75-100	349	16.8%	195	18.9%	139	13.4%
Urbanicity ^6*^	Village/rural	833	40%	416	40.3%	401	38.7%
	Small city/town (10,000-100.000)	462	22.2%	233	22.6%	242	23.4%
	Medium city/town (100,000-500.000)	306	14.7%	158	15.3%	140	13.5%
	Large city/town (over 500.000)	479	23%	226	21.9%	252	24.3%
First generation immigrant status ^7*^	No	476	22.9%	896	86.7%	936	90.4%
	Yes	48	2.3%	137	13.3%	99	9.6%
	Not Reported	1,556	74.8%	0	0%	0	0%
Region of residence (Federal State of Austria) ^8*^	Burgenland	82	3.9%	37	3.6%	35	3.4%
	Carinthia	132	6.3%	65	6.3%	64	6.2%
	Lower Austria	401	19.3%	196	19%	191	18.5%
	Salzburg	129	6.2%	62	6%	64	6.2%
	Styria	301	14.5%	145	14%	150	14.5%
	Tyrol	180	8.7%	89	8.6%	93	9%
	Upper Austria	334	16.1%	174	16.8%	168	16.2%
	Vienna	451	21.7%	219	21.2%	227	21.9%
	Vorarlberg	70	3.4%	46	4.5%	43	4.2%
Employment ^9*^	No	887	42.6%	403	39%	475	45.9%
	Yes	1,193	57.4%	630	61%	560	54.1%
Employed in health profession ^10*^	No	1,055	50.7%	560	54.2%	500	48.3%
	Yes	138	6.6%	70	6.8%	60	5.8%
	Not Reported	887	42.6%	403	39%	475	45.9%
Mental Health ^11*^	No	1,745	83.9%	851	82.4%	849	82%
	Yes	335	16.1%	182	17.6%	186	18%
Physical Health ^12*^	Yes	1,107	53.2%	566	54.8%	566	54.7%
	No	973	46.8%	467	45.2%	469	45.3%
Covid Test ^13*^	Negative	127	6.1%	823	79.7%	642	62%
	Positive	5	0.2%	46	4.5%	283	27.3%
	Not Reported	1,948	93.7%	164	15.9%	110	10.6%
COVID-19-related loss ^14*^	No	2,052	98.7%	965	93.4%	966	93.3%
	Yes	28	1.3%	68	6.6%	69	6.7%
Obesity ^15*^	BMI <30	1,641	78.9%	824	79.8%	818	79%
	BMI 30+	430	20.7%	199	19.3%	213	20.6%
	Not Reported	9	0.4%	10	1%	4	0.4%

COH-FIT questions: ^*^“How old are you? (in years)”, ^2*^“What is your gender?”, ^3*^“What race/ethnicity do you feel applies the most to you?”, ^4*^“What is your highest educational degree?”, ^5*^“How would you rate your socio-economic-status? (To respond, click on the bar and drag it with your mouse or finger)” (from 0=extremely below average to 100=extremely above average), ^6*^“What best describes the place where you live?”, ^7*^Contingent item: “What country/territory do you live in now? Over your lifetime, how many years have you lived in this country? In which country were you born?”, ^8*^“Please select your region”, ^9*^“Do you currently have a paid job?”, ^10*^Contingent item: “Do you have currently a paid job? Do you work in healthcare? (might include medical doctors, nurses, medical or nursing students, psychologists, social workers/case managers, other)”, ^11*^“Have you ever been diagnosed with a mental health condition by a doctor or psychologist?”, ^12*^“Have you ever been diagnosed with a medical disease by a health care professional?”, ^13*^Contingent item: if responded >0 on: “How many times have you been tested for COVID-19? Did you test positive?”, ^14*^“Has anyone close to you died because of COVID-19?”, ^15*^“Please enter your height (in centimeters), Please enter your current weight (in kilograms)”, obesity is calculated via body mass index.

### 3.2 Missing data

Missing data were <0.7% for all items except for three variables: having healthcare worker employment (43%), having had a COVID-19 infection (54%) and having first-generation immigrant status (37%) (**Supplementary Figure 1**). Missing values were not imputed for these three variables as they were contingent items only to be completed upon certain conditions (e.g., being in employment or having had a COVID-19 test). For all remaining items, data were imputed. Given that the data were collected using a probability-based sampling design by Gallup to ensure national representativeness, and the proportion of missing data in the analyzed variables was very low (<0.7%), survey weights were not applied in the present analyses.

### 3.3 WHO-5 well-being index

The WHO-5 well-being score decreased overall by 7.5 ± 17.7 points from pre-pandemic baseline (73.2 ± 19.7) to intra-pandemic (65.7 ± 24.1) (paired t=27.16, Mean Difference (MD)= 7.5 [95% CI 6.96, 8.03], p<.001, effect size d=.34). The intra-pandemic overall number is below 70, the mean well-being score found in general population studies (21). The proportion of individuals scoring <50, indicating depression, increased from 12.6% pre-pandemic baseline to 25.1% intra-pandemic (McNemar’s χ2 = 362.11, p<.001), with the proportion scoring <29, indicating clinical, major depression, increasing from 3.3% pre-pandemic baseline to 9.7% intra-pandemic (McNemar’s χ2 = 214.81, p<.001).

**Figure 2:**
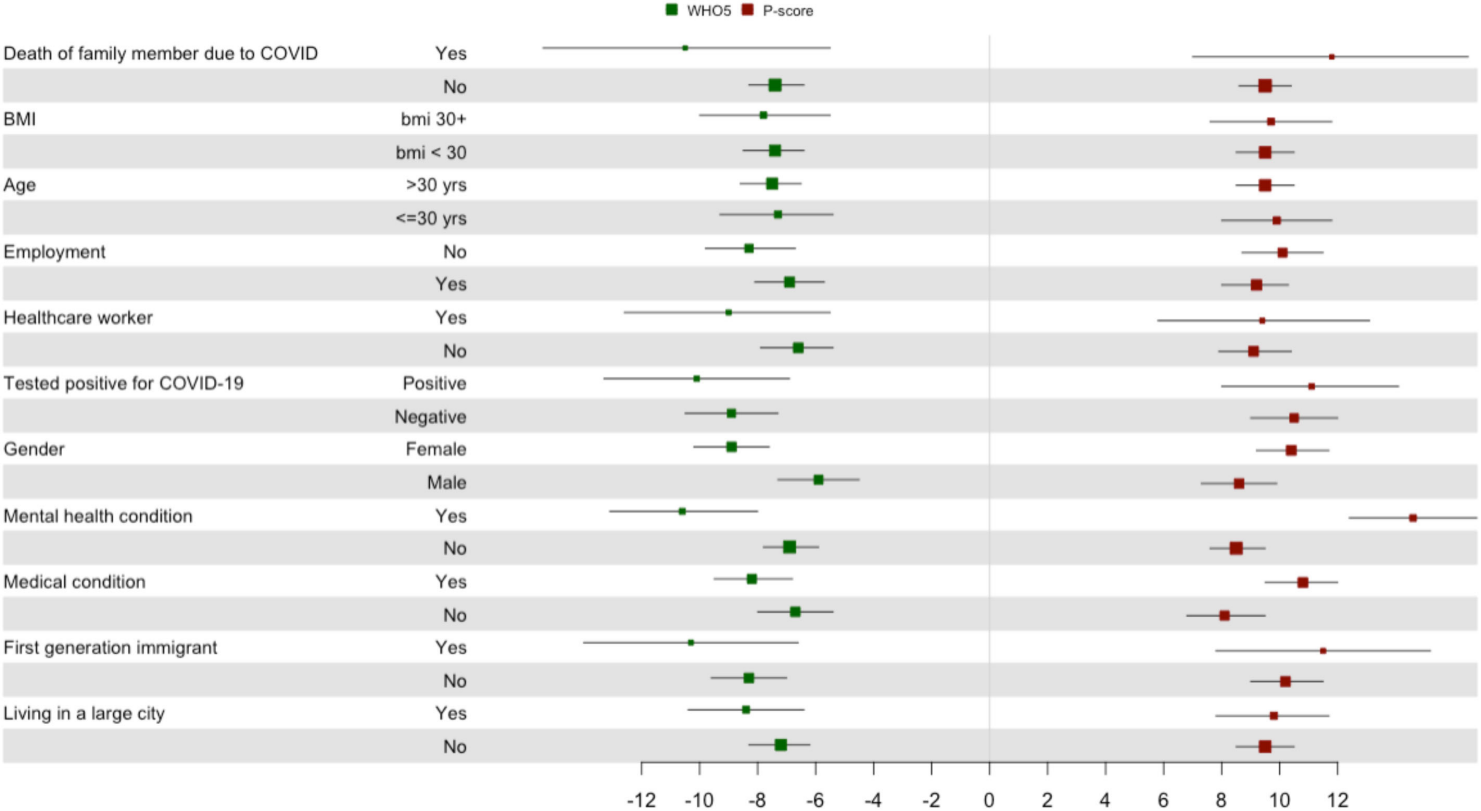
Forest plot of mean differences in well-being and ‘P-score’ changes across risk factors in adults (pooled across three survey waves to provide overall estimates). Forest plot of eleven defined risk groups (left column, yes/no) and changes in WHO-5 well-being score (green) and ‘P-score’ (red), x-axis with actual points of change: All higher risk groups exhibit greater decreases in well-being than their companion low-risk groups, but these greater decreases reach significance only in four risk groups: those without employment, females, those with a mental health condition, and those with a physical health condition (*). All higher risk groups have a higher ‘P-score’ than their companion low-risk groups, but these increases are only significant in three risk groups: those without employment, those with mental health condition, and those with a physical health condition.

With respect to at risk groups, all higher risk groups exhibit greater decreases in WHO-5 Well-Being Index than their companion low-risk groups. But these greater decreases reached statistical significance only in those without employment, females, those with a mental health condition and those with a physical health condition (**Figure 2**; **Supplementary Table 2**).

### 3.4 ‘P-score’

‘P-scores’ increased overall by 9.6 ± 15.0 points from a pre-pandemic baseline of 24.1 ± 19.5 to 33.7 ± 22.4 intra-pandemic (paired t=41.03, MD = 9.60 [95% CI 9.10, 10.02], p<.001, effect size d=.46). In the context of at-risk groups, significant increases in the ‘P-score’ were observed in individuals with mental health conditions, physical health conditions, and individuals who were unemployed (**Figure 2**; **Supplementary Table 2**).

#### 3.4.1 ‘P-score’ dimensional structure

**Figure 3:**
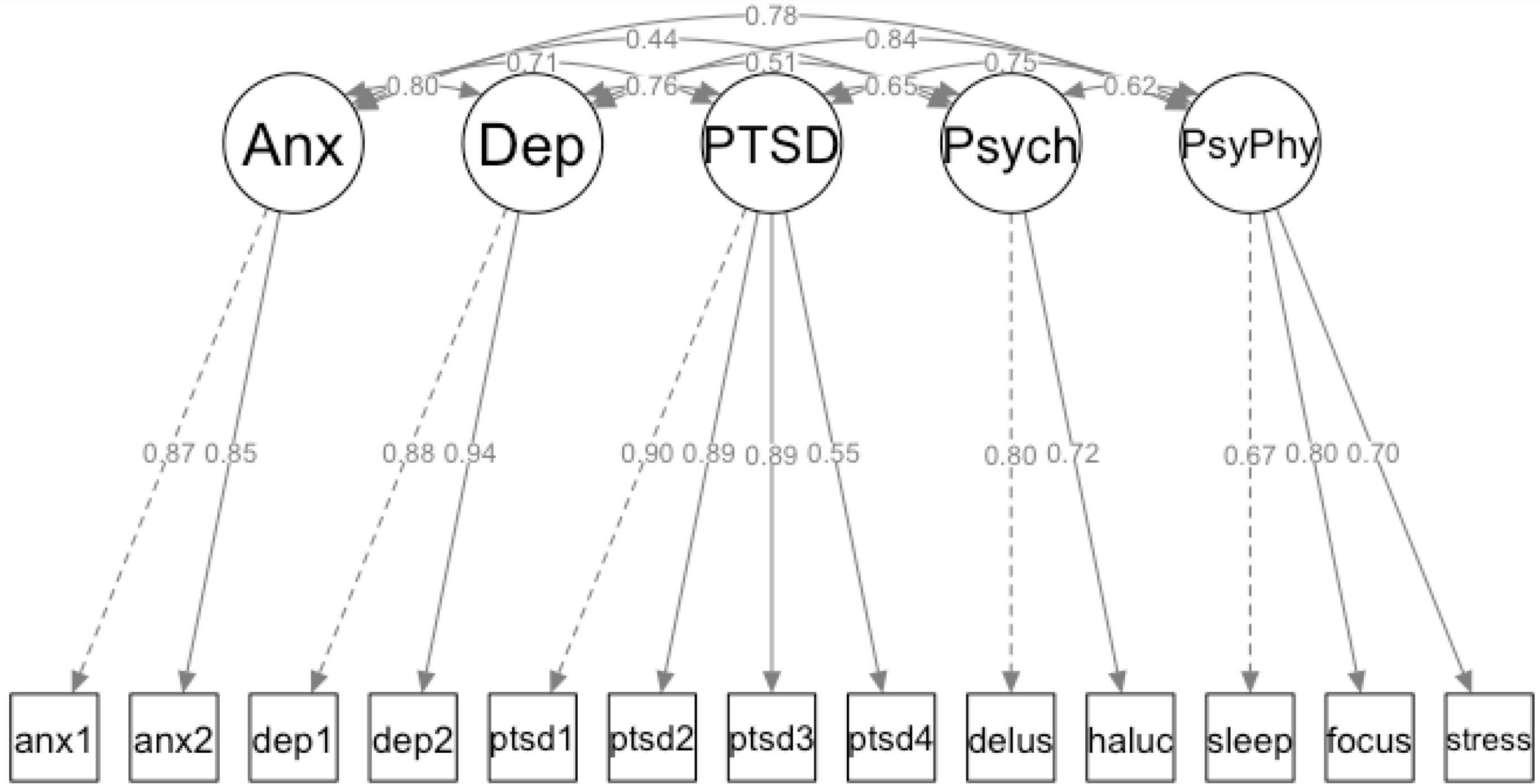
Sizeable correlations across domains supporting the computation of an overall ‘P-score’ (pooled across three survey waves). Factor structure of the composite psychopathology ‘P-score’ model showing good fit by confirmatory factor analysis (5 factors: anxiety, depression, post-traumatic stress disorder, psychosis, psychophysiologic). Anx, anxiety; Dep, depression; PTSD, post-traumatic stress disorder; Psych, psychosis (delus, haluc); PsyPhy, psychophysiology (sleep, focus, stress).

Overall, the correlated 5-factor ‘P-score’ model demonstrated a good fit, with CFI = 0.99, SRMR = 0.039, and RMSEA = 0.02. All item loadings were statistically significant and ranged from 0.70 – 0.94, indicating good convergent validity. Sizeable correlations across domains support the computation of an overall ‘P-score’ (**Figure 3**). Internal consistency was high for the overall measure (ω=0.94) and the five individual domains (from ω=0.74 for psychosis to ω=0.91 for depression).

### 3.5 Coping strategies

The five coping strategies most frequently rated as “very important” were internet use (69.9%), exercise and walking (62.9%), media use (TV, movies, radio, music) (55.0%), social media use and remote social interactions (48.2%) and meaningful hobby (47.3%). Importance ratings for the sixteen coping strategies are shown in **Table 2**; **Supplementary Figure 2**).

**Table 2:** The percentage of survey respondents rating the importance of each coping strategy during the pandemic (pooled across three survey waves).

Coping strategy	Not important	Somewhat important	Very important
Internet use	4.0	26.1	69.9
Exercise and walking	8.7	28.4	62.9
Media (TV, movies, radio, music)	7.3	37.7	55.0
Social media use/remote social interactions	11.9	39.9	48.2
Meaningful hobby	12.1	40.6	47.3
Keeping informed about the COVID-19 pandemic	10.3	43.5	46.2
Direct personal contact/ social contact or interactions	14.2	40.8	44.9
Spending time with a pet	42.2	23.4	34.5
Physical intimacy/sexual activity	24.0	44.4	31.6
Prescribed medications	41.2	28.1	30.7
Work (on site or from home)	33.1	37.9	29.0
Gaming	33.0	41.0	26.0
Studying or learning something new	35.9	44.0	20.1
Religion/meditation/spirituality	57.6	28.4	14.0
Other	50.4	38.8	10.7
Use of substances (tobacco, alcohol, other)	62.0	28.7	9.3

### 3.6 WHO-5 well-being and ‘P-score’ change across survey waves

**Figure 4:**
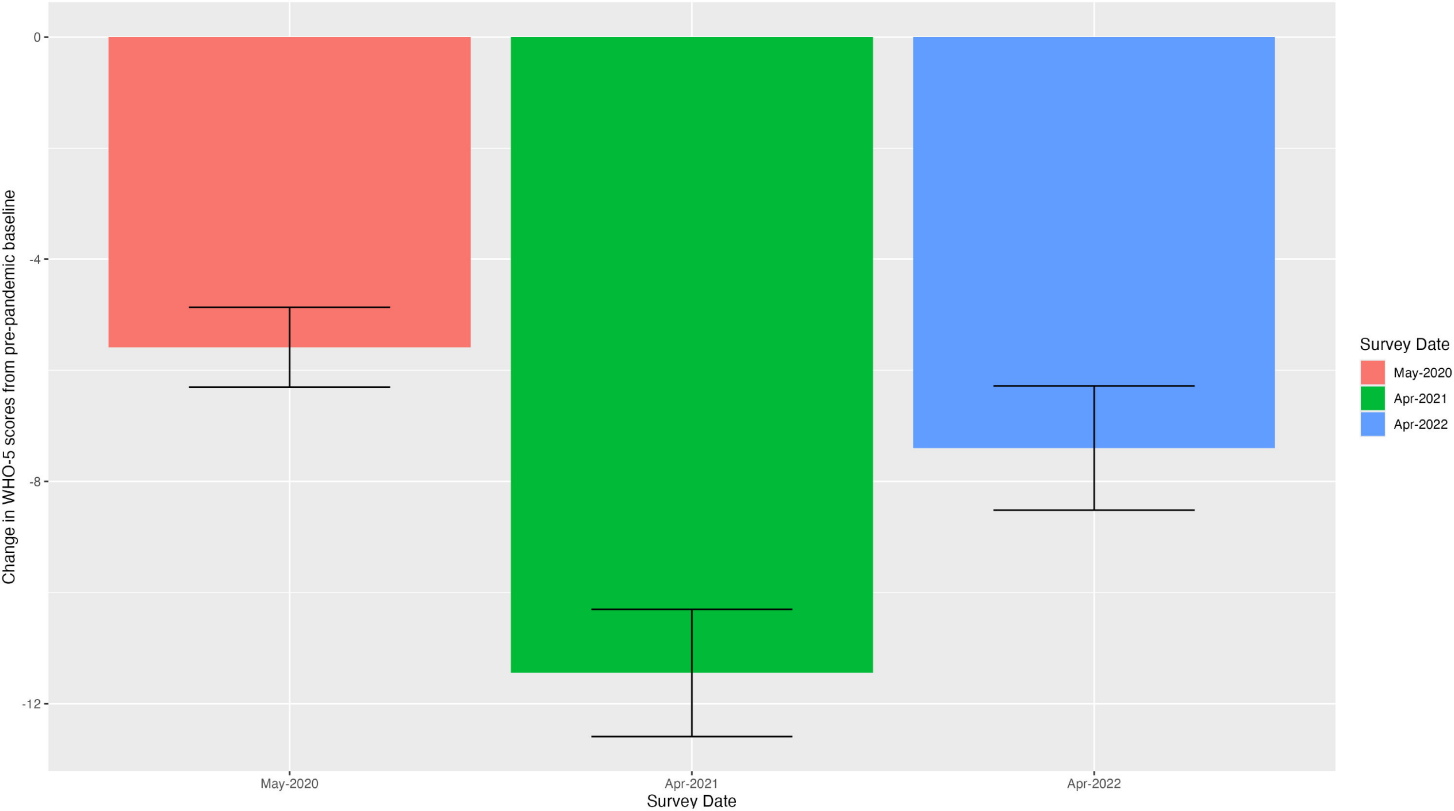
WHO-5 mean well-being score reduction from baseline across the three different survey waves. Error bars represent standard errors.

**Figure 5:**
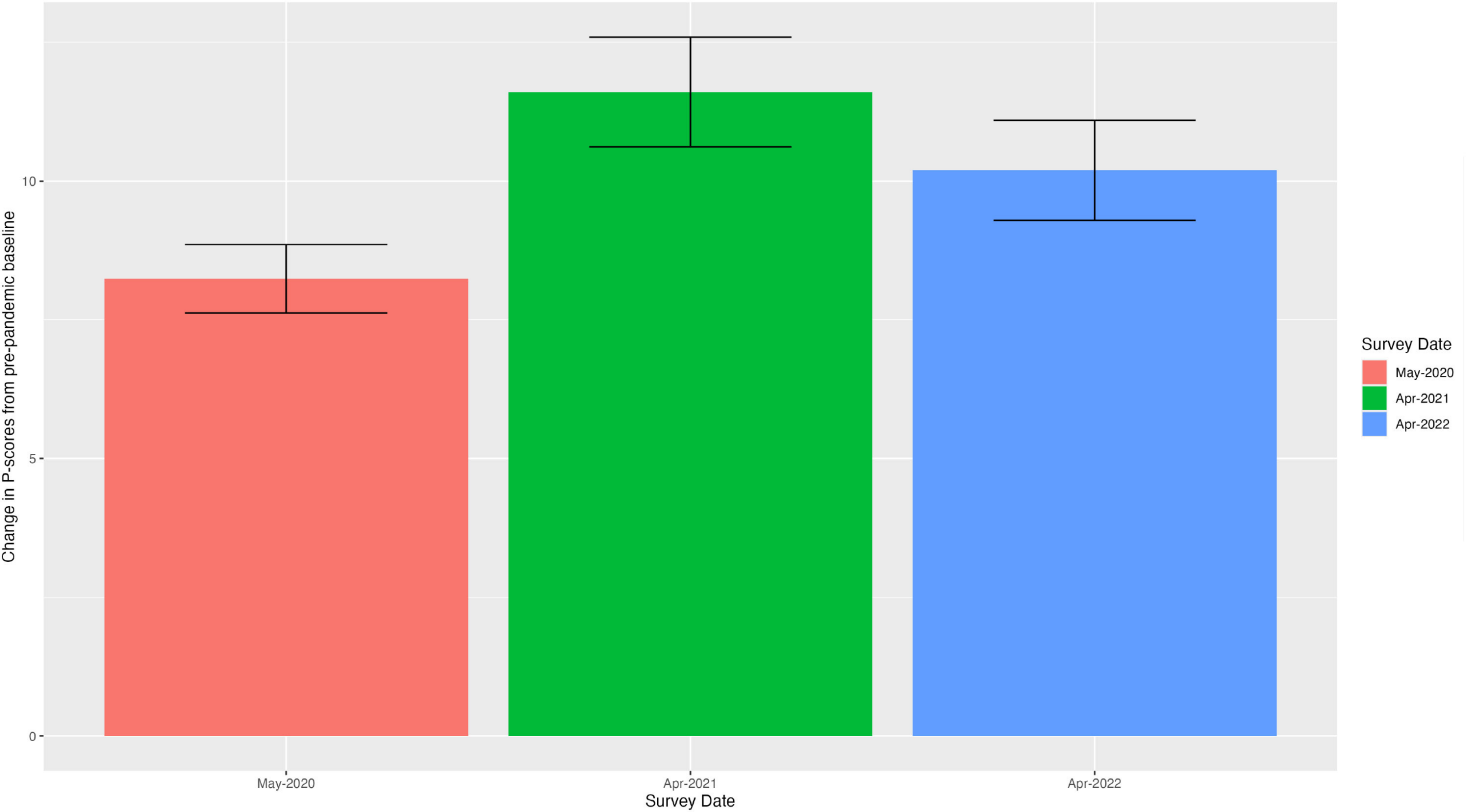
Mean ‘P-score’ increase from baseline across the three different survey waves. Error bars represent standard errors.

ANOVA showed significant differences in the extent of WHO-5 well-being reduction from pre-pandemic baseline across the three different survey waves. Mean changes from pre-pandemic baseline for Wave 1 (May 2020) = -5.59, points, Wave 2 (April 2021) = -11.40 points and Wave 3 (April 2022) = -7.40 points, Overall, a significant change from pre-pandemic baseline was observed, F (2, 4145) = 38.18, p<.001 (see **Figure 4**). Planned contrasts showing a greater decrease from pre-pandemic baseline in the Wave 2 mid-pandemic period in April 2021 compared to Wave 1 May 2020 (t=8.74, MD = -5.81 [95% CI -4.54, -7.17], p <.001) and Wave 3 April 2022 (t=4.05, MD = -4.00 [95% CI -2.52, -5.56], p <.001) periods. WHO-5 well-being score still had not returned to levels observed in May 2020 (see **Figure 4**). ANOVA showed similar differences in P-score change from pre-pandemic baseline across survey waves, F(2, 4145) = 18.74, p <.001 (see **Figure 5**), with mean changes from pre-pandemic baseline for Wave 1 (May 2020) = 8.24 points, Wave 2 (April 2021) = 11.6 points and Wave 3 (April 2022) = 10.2 points Contrasts showing a greater increase during the mid-pandemic April 2021 period compared to the May 2020 (t=5.92, MD = 3.36 [95% CI 2.25, 4.48], p <.001) and April 2022 (t=2.15, MD = 1.41 [95% CI 0.12, 2.69], p <.001) periods, but as demonstrated, in April 2022, the ‘P-score’ remained high and still had not returned to the level observed in May 2020 (see **Figure 5**).

### 3.7 Evaluation of recall bias

ANOVA contrasts comparing pre-pandemic baseline WHO-5 well-being scores revealed that retrospectively recalled pre-pandemic baseline WHO-5 well-being scores did not significantly differ for Wave 2 compared to Wave 1, MD = 0.69 [95% CI = -0.77, 2.15], p=.356, but were significantly lower at Wave 3 compared to Wave 1, MD=-2.97 [95% CI = 1.51, 4.43], p<.001. No difference was found for baseline ‘P-scores’ when comparing Wave 1 with either Wave 2 (MD=-0.15, [95% CI = -1.61, 1.30], p=.84) or Wave 3 (MD = 0.64, [95% CI = -0.81, 2.09], p=.39) pre-pandemic baseline scores.

## 4 Discussion

### 4.1 Summary of study results

This repeated cross-sectional study assessed the impact of the COVID-19 pandemic on quality of life and psychopathology in the Austrian general population of adults across three time points (May 2020 – April 2022). Seven principal findings emerged: i) Significant deterioration in both well-being and mental health indicators were observed over the study period; ii) The most pronounced decline in well-being (mean change > 11 points) occurred in mid-pandemic (April 2021); iii) The most pronounced increase in ‘P-score’ (mean change > 11 points) occurred in mid-pandemic (April 2021); iv) Mental health and well-being indicators remained impaired compared with pre-pandemic baselines within each of the 2-weeks of intra-pandemic time period, even in the last survey wave (Wave 3), when the severity of the pandemic had already attenuated and in the overall COH-FIT study the population had adapted in the response to the pandemic with a return of the WHO-5 and ‘P-score’ to pre-pandemic baseline levels (9, 19). Well-being and mental-health indices had not returned to pre-pandemic baseline values or values at wave one in May 2020 two years after the initial outbreak (well-being decrease mean > 7 points and ‘P-score’ increase mean > 10 points); v). The prevalence of depressive symptoms within the Austrian COH-FIT sample approximately doubled during the two-year period, while the prevalence of clinically significant major depressive disorder tripled during the two-year period, using cutoffs for these outcomes published previously (20, 22); vi) Groups at highest risk for poor well-being and mental health outcomes included women, individuals with pre-existing mental or physical health conditions, and those without employment; and vii) The most important coping strategies were internet use, physical exercise, traditional media consumption, social media use and remote interactions and engagement in meaningful hobbies.

### 4.2 Comparison with international evidence

The Austrian findings mirror global reports of pandemic-related declines in mental health (5, 9). Nevertheless, the international literature is heterogenous as demonstrated by some examples: Salanti et al. (4) analyzed 41 international studies across seven mental health outcomes and found increased odds of psychological distress, depression, and anxiety during the first two months of the pandemic (odds ratios 1.23 - 2.08), particularly where and when infection rates, mortality, and restrictive measures were high. An analysis of the large multi-wave COH-FIT study from Greece (26) found significant reductions in well-being and increases in overall psychopathology across intra-pandemic assessment phases, particularly during lockdown periods. This finding highlights the negative impact of restrictive measures. While this result is consistent with our findings of marked mid-pandemic deterioration in Austria, the Greek COH-FIT data emphasize more acute effects with stabilization thereafter. In contrast, our results in the web-active Austrian general public indicate more persistent impairments without full recovery to pre-pandemic levels.

Individual study results, however, ranged from substantial deterioration to no detectable change. Lok et al. (8) meta-analyzed nationally or regionally representative European samples assessed at least twice up to early 2022. In contrast to our data, that review found no overall change in the prevalence of emotional distress, anxiety, or depression. Yet, it still reported small increases in self-reported emotional distress associated with stricter school closures and social-distancing measures, mirroring patterns seen in Austria. Moreover, both meta-analyses highlighted large heterogeneity between individual study results which could be due to differences in study methodology, assessment, population selection, individual time points of assessment and relationship to regionally and nationally variable restriction measures. An analysis of the COH-FIT data from Switzerland (27) identified heterogeneous well-being trajectories. Most individuals remained resilient, while a minority experienced a decline in well-being or had persistently low well-being. While this pattern aligns with our identification of vulnerable subgroups, it contrasts with our findings, as the Swiss data suggest greater overall resilience and more distinct subgroup trajectories, rather than sustained, population-wide residual impairment as found in Austria. A meta-analysis by Sun et al., 2023 (28) concluded that the pandemic had minimal impact on mental health - an interpretation that diverges from our findings. The authors suggest caution in interpreting results because of high risk of bias in many studies and substantial heterogeneity. Methodological diversity - including differences in sampling, assessment tools, survey timing, and regional policy responses - likely contributes to these discrepancies.

### 4.3 Unique contribution of the Austrian COH-FIT study

Our repeated cross-sectional, population-representative design tracked trajectories from May 6^th^ 2020 through April 25^th^ 2022, thereby extending beyond the short-term horizons of many prior meta-analyses. Notably, while the global COH-FIT sample (N = 121,066; Solmi et al., 2024 (9) showed a return of well-being and ‘P-scores’ to near pre-pandemic baseline levels after two years and also other meta-analyses showed only transient or minimal psychological consequences, the Austrian representative cohort did not seem to fully returned to pre-pandemic baseline with regards to WHO-5 and ‘P-score’ ratings. While these results could be affected by a focus on the web-active general population, repeated cross-sectional design, and retrospectively rated pre-pandemic baseline values, these results indicate a need for sustained mental-health surveillance and intervention in Austria.

### 4.4 Implications

The findings of the present study are broadly consistent with the systematic review by Dragioti et al., 2022 (5), which only reviewed publications until September 2020. Our results suggest that the pandemic may be associated with elevated rates of mental-health morbidity across the population. Specific subgroups – e.g., unemployed individuals, people with somatic or mental disorders, and females – may be disproportionately affected. These findings still highlight the need for public-health planning that focuses on targeted preventive measures for high-risk groups, expanding evidence-based coping-strategy programs, and long-term psychosocial surveillance to support resilience and reduce potential lasting psychological harm.

### 4.5 Austrian COH-FIT findings in the context of national research

Our results align with a representative cross-sectional study comparing Austrian adults surveyed in April 2020 (N = 1,005) and spring 2022 (N = 1,031), which reported persistently elevated rates of mental-health problems two years after the pandemic’s onset, despite only minimal restrictions at the later time point (11). One plausible explanation is that psychological symptoms recede more slowly than they emerge. The succession of stringent lockdowns implemented in Austria through May 2021 may have left lasting psychological consequences. Additional uncertainty regarding the pandemic’s long-term trajectory likely intensifies distress. Even after widespread vaccination, it became evident that breakthrough infections and long-COVID effects remained possible (29), and that secondary consequences - such as rising energy costs - would further undermine economic stability and quality of life. While such stressors were common globally, meta-analytic evidence suggests that downstream mental-health effects have varied considerably by region, underscoring the need for detailed cross-national comparisons.

The findings of another Austrian study by Oberndorfer et al. (12), which collected data over 30 waves from March 2020 to March 2022 and involved 3,293 participants, differed from our results. All components of subjective well-being (SWB) – negative affect, positive affect, and life satisfaction – fluctuated throughout the COVID-19 pandemic, however, the magnitude of these changes was small. One possible explanation for this discrepancy may be that Oberndorfer et al. (2022) included participants aged 14 years and older in their study. Additionally, different well-being measures were used, which may have contributed to the differing results.

### 4.6 Vulnerable groups and coping strategies

Deterioration in both well-being and psychopathology was consistently greater among women, individuals with pre-existing mental or physical disorders, and those who were unemployed. These risk patterns replicate earlier Austrian work identifying young adults (< 35 years), women, and individuals affected by job loss or low income as particularly vulnerable (11, 13, 15, 30). Women may have experienced a disproportionate burden due to their higher baseline prevalence of mental disorders (GBD Collaborators, 2022) and increased responsibility related to home-schooling, childcare, and domestic duties during lockdowns (31). Unemployment - already a well-established risk factor for depression (32) and poor general health (33) was further compounded by pandemic-induced financial hardship. A qualitative study in Western Austria involving 151 participants demonstrated that financial strain contributed to declines in physical and mental health, substandard living conditions, and social isolation, all of which were exacerbated during the pandemic (34). These converging findings highlight the need for public-health interventions that explicitly prioritize high-risk groups and address structural determinants of mental health. A Danish COH-FIT study analysis found that mental health declined more severely among individuals with a history of mental illness, although the overall effects showed heterogeneity and partial improvement over time (35). Regarding adaptative strategies, Austrian respondents most frequently identified internet use, physical exercise, traditional and social-media engagement, remote interactions, and participation in meaningful hobbies as helpful - mirroring results from the global COH-FIT findings (9) and from the COH-FIT sample in Greece (36). Physical activity is a well-established intervention for both physical and mental health (37). While excessive media and social-media use can be detrimental under normal conditions (38), during lockdowns these platforms offered essential channels for information and social connection. Clarifying which digital and behavioral strategies most effectively mitigate psychological stress under pandemic conditions remains crucial for future preparedness.

### 4.7 Strengths and limitations

A key strength of this study lies in its use of data from a relatively large and representative sample of Austrian adults. Moreover, the repeated cross-sectional design with three waves of assessment over a two-year period allowed for the analysis of temporal trends across distinct phases of the COVID-19 pandemic. This design enabled a more nuanced understanding of changes in well-being and psychopathology, as well as the potential directionality of associations between risk factors and outcomes. The use of validated and standardized measures further strengthens the study’s methodological rigor and facilitates international comparability.

However, several limitations must be acknowledged. First, mental health outcomes were assessed via self-report questionnaires rather than clinician-administered interviews, which may have introduced subjective bias. Second, the use of computer-assisted web interviewing (CAWI) method for the representative sample likely excluded individuals without internet access or sufficient digital literacy, potentially limiting the generalizability of the results to digitally connected populations. Third, baseline mental health prior to the pandemic was assessed retrospectively rather than measured directly, raising concerns regarding the comparability of change estimates across waves. As recall distances increased with each subsequent wave, the reported change scores provide valuable indications of perceived change over time, though they should be interpreted with caution as reflecting subjective retrospective appraisal rather than changes from a directly measured baseline, and are therefore considered exploratory in nature. While baseline WHO-5 well-being scores did not differ significantly between Wave 1 and Wave 2, they were significantly lower at Wave 3, suggesting that recall bias may have contributed to the apparent worsening of well-being at that time point. Nonetheless, analyses of ´P-score´ trajectories across all three time points revealed consistent patterns without systematic drift, mirroring findings from the international COH-FIT dataset (9). Fourth, the analyses presented here focus on the identification of high-risk groups rather than on exploring the underlying influences on these associations, and that the observed associations were not adjusted for potential confounding factors. Additional analyses to examine the possible influence of confounders, moderators and mediators on these associations using a causal analysis approach are currently underway and will be reported in future publications. Lastly, while this study extends beyond the time frame covered by most meta-analyses e.g. (4, 8, 28), it does not capture developments beyond April 2022, and thus cannot reflect more recent changes in population mental health.

### 4.8 Conclusion

In summary, the COVID-19 pandemic had a sustained and significant negative impact on the well-being and mental health of the Austrian general population. The prevalence of depressive symptoms approximately doubled and that of clinically significant major depressive disorder tripled. This substantial increase reflects the profound psychological impact of the pandemic, including social isolation, economic insecurity, and prolonged uncertainty about the future - all major contributors to mental distress. Vulnerable subgroups - particularly women, individuals with pre-existing mental or physical health conditions, and the unemployed - experienced disproportionate psychological deterioration, with the most severe impact occurring during the mid-pandemic period. Notably, and while acknowledging the exploratory nature of retrospective change estimates, mental health indicators had not returned to pre-pandemic baseline levels even two years after the initial outbreak.

We further contextualize these international findings in the Conclusions section. Taken together and viewed in the context of the existing literature, including across different COH-FIT study country samples that were assessed with identical measures, these results highlight that there are differences in effects of the COVID-19 pandemic across different countries and cultures and reactions to the pandemic that may get lost in multinational studies and meta-analyses. Moreover, these results underscore the urgent need for proactive, country specific targeted public-health strategies. Policy makers should use these insights to identify and protect high-risk groups, ensure access to effective coping resources, and implement timely, evidence-based mental-health support during future public health crises. It is essential that public health interventions and crisis management are guided not only by medical and logistical coordination but also by empathy, respect and a comprehensive understanding of psychological and social well-being. Furthermore, individuals still experiencing lingering psychological effects should be systematically identified and provided with appropriate, evidence-based care to prevent long-term psychiatric morbidity in the post-pandemic period.

## Data Availability

The datasets presented in this article are not readily available because the primary data are not publicly available because permission for data sharing beyond the study personnel was not included in the written informed consent form. Requests to access the datasets should be directed to Not applicable.

## References

[1] Chiou P Yoch M . Long COVID is a serious health concern in Europe. Institute For Health Metrics Eval. (2023).

[2] Prati G Mancini AD . The psychological impact of COVID-19 pandemic lockdowns: a review and meta-analysis of longitudinal studies and natural experiments. Psychol Med. (2021) 51:201–11. doi: 10.1017/s0033291721000015. PMID: 33436130 PMC7844215

[3] Robinson E Sutin AR Daly M Jones A . A systematic review and meta-analysis of longitudinal cohort studies comparing mental health before versus during the COVID-19 pandemic in 2020. J Affect Disord. (2022) 296:567–76. doi: 10.1016/j.jad.2021.09.098. PMID: 34600966 PMC8578001

[4] Salanti G Peter NL Tonia T Holloway A Darwish L Kessler RC . Changes in the prevalence of mental health problems during the first year of the pandemic: a systematic review and dose-response meta-analysis. BMJ Ment Health. (2024) 27:1–8. doi: 10.1136/bmjment-2024-301018. PMID: 38876492 PMC11177678

[5] Dragioti E Li H Tsitsas G Lee KH Choi J Kim J . A large-scale meta-analytic atlas of mental health problems prevalence during the COVID-19 early pandemic. J Med Virol. (2022) 94:1935–49. doi: 10.1002/jmv.27549. PMID: 34958144 PMC9015528

[6] Lindqvist Bagge AS Lekander M Olofsson Bagge R Carlander A . Mental health, stress, and well-being measured before (2019) and during (2020) COVID-19: a Swedish socioeconomic population-based study. Psychol Health. (2024) 39:1787–804. doi: 10.1080/08870446.2023.2257747. PMID: 37728316

[7] Mao Z Pepermans K Beutels P . Relating mental health, health-related quality of life and well-being in the aftermath of the COVID-19 pandemic: a cross-sectional comparison in 14 European countries in early 2023. Public Health. (2025) 238:16–22. doi: 10.1016/j.puhe.2024.11.010. PMID: 39579613

[8] Lok V Sjoqvist H Sidorchuk A Flodin P Osika W Daly M . Changes in anxiety and depression during the COVID-19 pandemic in the European population: a meta-analysis of changes and associations with restriction policies. Eur Psychiatry: J Assoc Eur Psychiatrists. (2023) 66:e87. doi: 10.1192/j.eurpsy.2023.2467. PMID: 37881862 PMC10755582

[9] Solmi M Thompson T Estrade A Agorastos A Radua J Cortese S . Global and risk-group stratified well-being and mental health during the COVID-19 pandemic in adults: results from the international COH-FIT Study. Psychiatry Res. (2024) 342:115972. doi: 10.1016/j.psychres.2024.115972. PMID: 39305825

[10] Schurr T Frajo-Apor B Pardeller S Plattner B Tutzer F Schmit A . Overcoming times of crisis: unveiling coping strategies and mental health in a transnational general population sample during and after the COVID-19 pandemic. BMC Psychol. (2024) 12:493. doi: 10.1186/s40359-024-02001-3. PMID: 39300578 PMC11412033

[11] Humer E Schaffler Y Jesser A Probst T Pieh C . Mental health in the Austrian general population during COVID-19: cross-sectional study on the association with sociodemographic factors. Front Psychiatry. (2022) 13:943303. doi: 10.3389/fpsyt.2022.943303. PMID: 36506423 PMC9729349

[12] Oberndorfer M Stolz E Dorner TE . Evidence for changes in population-level subjective well-being during the COVID-19 pandemic from 30 waves of representative panel data collected in Austria between March 2020 and March 2022. Public Health. (2022) 212:84–8. doi: 10.1016/j.puhe.2022.09.004. PMID: 36265427 PMC9472574

[13] Pieh C Budimir S Probst T . The effect of age, gender, income, work, and physical activity on mental health during coronavirus disease (COVID-19) lockdown in Austria. J Psychosom Res. (2020) 136:110186. doi: 10.1016/j.jpsychores.2020.110186. PMID: 32682159 PMC7832650

[14] Dale R Budimir S Probst T Stippl P Pieh C . Mental health during the COVID-19 lockdown over the Christmas period in Austria and the effects of sociodemographic and lifestyle factors. Int J Environ Res Public Health. (2021) 18:1–15. doi: 10.3390/ijerph18073679. PMID: 33916019 PMC8036255

[15] Pieh C Budimir S Humer E Probst T . Comparing mental health during the COVID-19 lockdown and 6 months after the lockdown in Austria: a longitudinal study. Front Psychiatry. (2021) 12:625973. doi: 10.3389/fpsyt.2021.625973. PMID: 33859579 PMC8042148

[16] Solmi M Estrade A Thompson T Agorastos A Radua J Cortese S . The collaborative outcomes study on health and functioning during infection times in adults (COH-FIT-Adults): design and methods of an international online survey targeting physical and mental health effects of the COVID-19 pandemic. J Affect Disord. (2022) 299:393–407. doi: 10.1016/j.jad.2021.07.048. PMID: 34949568 PMC8288233

[17] Solmi M Estrade A Thompson T Agorastos A Radua J Cortese S . Physical and mental health impact of COVID-19 on children, adolescents, and their families: the Collaborative Outcomes study on Health and Functioning during Infection Times - Children and Adolescents (COH-FIT-C&A). J Affect Disord. (2022) 299:367–76. doi: 10.1016/j.jad.2021.09.090. PMID: 34606810 PMC8486586

[18] Solmi M Thompson T Estrade A Agorastos A Radua J Cortese S . Validation of the Collaborative Outcomes study on Health and Functioning during Infection Times (COH-FIT) questionnaire for adults. J Affect Disord. (2023) 326:249–61. doi: 10.1016/j.jad.2022.12.022. PMID: 36586617 PMC9794522

[19] Solmi M Thompson T Cortese S Estrade A Agorastos A Radua J . Collaborative outcomes study on health and functioning during infection times (COH-FIT): insights on modifiable and non-modifiable risk and protective factors for wellbeing and mental health during the COVID-19 pandemic from multivariable and network analyses. Eur Neuropsychopharmacol. (2025) 90:1–15. doi: 10.1016/j.euroneuro.2024.07.010. PMID: 39341043

[20] Topp CW Ostergaard SD Sondergaard S Bech P . The WHO-5 Well-Being Index: a systematic review of the literature. Psychother Psychosom. (2015) 84:167–76. doi: 10.1159/000376585. PMID: 25831962

[21] Ellervik C Kvetny J Christensen KS Vestergaard M Bech P . Prevalence of depression, quality of life and antidepressant treatment in the Danish General Suburban Population Study. Nord J Psychiatry. (2014) 68:507–12. doi: 10.3109/08039488.2013.877074. PMID: 24476587

[22] Birket-Smith M Hansen BH Hanash JA Hansen JF Rasmussen A . Mental disorders and general well-being in cardiology outpatients--6-year survival. J Psychosom Res. (2009) 67:5–10. doi: 10.1016/j.jpsychores.2009.01.003. PMID: 19539812

[23] Salazar de Pablo G Vaquerizo-Serrano J Catalan A Arango C Moreno C Ferre F . Impact of coronavirus syndromes on physical and mental health of health care workers: systematic review and meta-analysis. J Affect Disord. (2020) 275:48–57. doi: 10.1016/j.jad.2020.06.022. PMID: 32658823 PMC7314697

[24] Buuren SV Groothuis-Oudshoorn K . mice: multivariate imputation by chained equations in R. J Stat Softw. (2010) 45(i03):1–68. doi: 10.32614/cran.package.mice

[25] Brown TA . Confirmatory factor analysis for applied research. London: Guilford Press (2006).

[26] Agorastos A Christogiannis C Mavridis D Seitidis G Kontouli KM Tsokani S . Impact of COVID-19 pandemic-related restrictive measures on overall mental and physical health and well-being, specific psychopathologies and emotional states in representative adult Greek population: results from the largest multi-wave, online national survey in Greece (COH-FIT). Psychiatry Res. (2025) 348:116479. doi: 10.1016/j.psychres.2025.116479. PMID: 40179637

[27] Ehrler M Vogt A Eichelberger D Greutmann M Hagmann CF Jenni OG . Psychological well-being in adults across the COVID-19 pandemic: a two-year longitudinal study. Int J Public Health. (2025) 70:1608347. doi: 10.3389/ijph.2025.1608347. PMID: 40786604 PMC12331532

[28] Sun Y Wu Y Fan S Dal Santo T Li L Jiang X . Comparison of mental health symptoms before and during the covid-19 pandemic: evidence from a systematic review and meta-analysis of 134 cohorts. BMJ. (2023) 380:e074224. doi: 10.1136/bmj-2022-074224. PMID: 36889797 PMC9992728

[29] Lopez Bernal J Gower C Andrews N Public Health England Delta Variant Vaccine Effectiveness Study G . Effectiveness of covid-19 vaccines against the B.1.617.2 (Delta) variant. Reply. N Engl J Med. (2021) 385:e92. doi: 10.1056/nejmoa2108891. PMID: 34758250

[30] Dale R O'Rourke T Humer E Jesser A Plener PL Pieh C . Mental health of apprentices during the COVID-19 pandemic in Austria and the effect of gender, migration background, and work situation. Int J Environ Res Public Health. (2021) 18:1–11. doi: 10.3390/ijerph18178933. PMID: 34501523 PMC8430826

[31] Racine N Hetherington E McArthur BA McDonald S Edwards S Tough S . Maternal depressive and anxiety symptoms before and during the COVID-19 pandemic in Canada: a longitudinal analysis. Lancet Psychiatry. (2021) 8:405–15. doi: 10.1016/s2215-0366(21)00074-2. PMID: 33773109 PMC8824360

[32] Kohler CA Evangelou E Stubbs B Solmi M Veronese N Belbasis L . Mapping risk factors for depression across the lifespan: an umbrella review of evidence from meta-analyses and Mendelian randomization studies. J Psychiatr Res. (2018) 103:189–207. doi: 10.1016/j.jpsychires.2018.05.020 29886003

[33] Wilson SH Walker GM . Unemployment and health: a review. Public Health. (1993) 107:153–62. doi: 10.1016/s0033-3506(05)80436-6. PMID: 8511234

[34] Kerschbaumer L Crossett L Holaus M Costa U . COVID-19 and health inequalities: the impact of social determinants of health on individuals affected by poverty. Health Policy Technol. (2024) 13:1–7. doi: 10.1055/s-0044-1794306. PMID: 3785596

[35] Vendsborg P Jarlstrup NS Hoffmann SH Nordentoft M Correll CU Solmi M . Health, psychological distress, and functioning during the COVID-19 pandemic among Danish adults with and without a preexisting mental illness. Int J Environ Res Public Health. (2025) 22:1–10. doi: 10.3390/ijerph22081260. PMID: 40869845 PMC12386791

[36] Agorastos A Christogiannis C Mavridis D Seitidis G Kontouli KM Tsokani S . Restrictive measures and course of behavioral outcomes in representative adult Greek population during the COVID-19 pandemic: results on functioning, coping, alcohol/substance abuse, smoking, gambling, pro-social activities and domestic violence from the largest multi-wave COVID-19 online national survey in Greece (COH-FIT). J Psychiatr Res. (2025) 190:480–9. doi: 10.1016/j.jpsychires.2025.07.014. PMID: 40886563

[37] Firth J Solmi M Wootton RE Vancampfort D Schuch FB Hoare E . A meta-review of "lifestyle psychiatry": the role of exercise, smoking, diet and sleep in the prevention and treatment of mental disorders. World Psychiatry. (2020) 19:360–80. doi: 10.1002/wps.20773. PMID: 32931092 PMC7491615

[38] Ahmed O Walsh EI Dawel A Alateeq K Espinoza Oyarce DA Cherbuin N . Social media use, mental health and sleep: a systematic review with meta-analyses. J Affect Disord. (2024) 367:701–12. doi: 10.1016/j.jad.2024.08.193. PMID: 39242043

