# Supplementary material for: Two years of COVID-19: persistently reduced well-being and increases in global psychopathology during the pandemic in a representative Austrian population-sample within the COH-FIT study
Source: Front Psychiatry. 2026 Jun 1;17:1783600. doi: 10.3389/fpsyt.2026.1783600 (PMC13265464; doi:10.3389/fpsyt.2026.1783600)

Supplementary Material

**Supplementary Table 1. The percentage of respondents in each category (gender, having a paid job, age, highest educational degree) in the sampled data in the study compared to general population statistics of Austria (December 2020). The numbers and comparisons are consistent with the representative sampling method used in the study.**

| **category** | **Austrian general population statistics (>18 years) (%)** | **sampled data in current study (%)** |
| --- | --- | --- |
| male gender ^*^ | 48.80 | 47.42 |
| female gender ^*^ | 51.20 | 52.58 |
| having no paid job | 43.50 | 42.56 |
| having a paid job | 56.50 | 57.44 |
| age 18-29 years | 17.40 | 18.23 |
| age 30-49 years | 32.60 | 34.04 |
| age >50 years | 50.00 | 47.73 |
| no educational degree | 0.30 | 0.22 |
| Primary school degree | 0.80 | 0.97 |
| High school degree | 62.5 | 73.1 |
| College/university degree | 35.2 | 24.6 |
| PhD | 1.20 | 1.11 |
| N | 8,877,067 | 4,148 |

^*^ Population reference statistics do not contain population values for non-binary categories

**Supplementary Figure 1. Item completion rate in the survey in Austria shown on y-axis (in percent) and different variables shown on x-axis in survey order.**


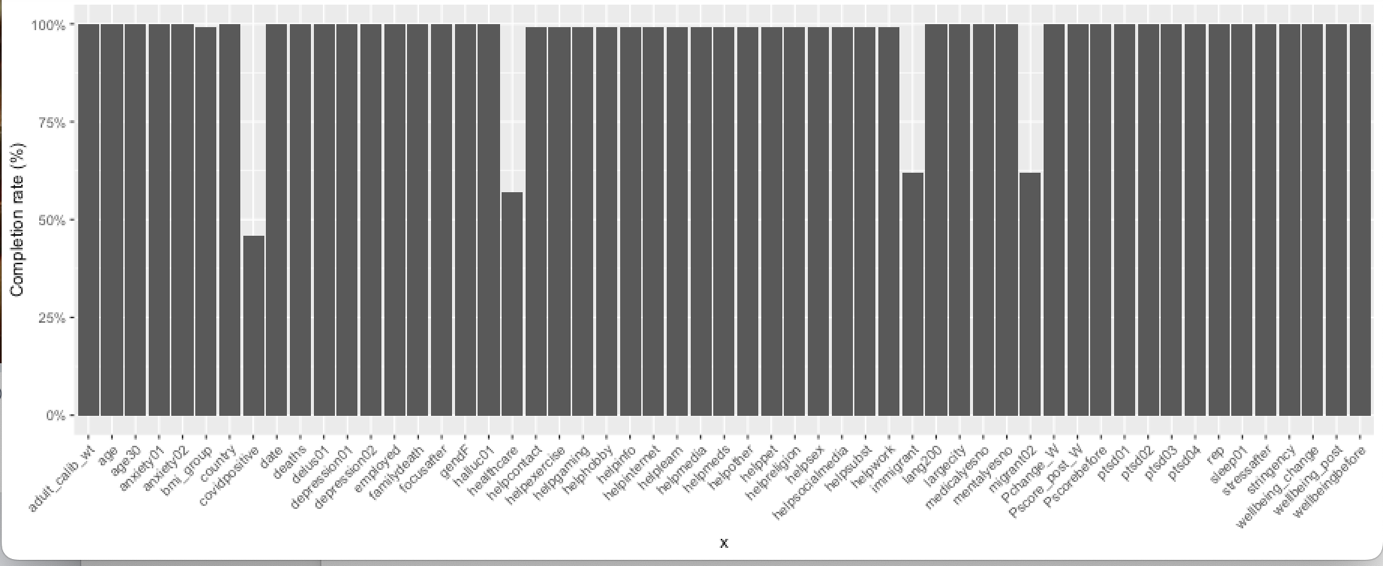


## 2

## Supplementary Table 2: Change in WHO-5 well-being scores and ‘P-scores’ across individuals at risk of poor outcomes during the COVID-19 pandemic. Risk groups were previously described in literature. All higher risk groups exhibit greater decreases in well-being than their companion low-risk groups, but decreases reaching significance only in those without employment, females, those with a mental health condition, and those with a physical health condition. All higher risk groups have a higher ‘P-score’ than their companion low-risk groups, but significant increases in the ‘P-score’ were observed only in individuals who were unemployed, individuals with mental health conditions, and individuals with physical health conditions.

|  | | | | **Pre-pandemic** | | **During pandemia** | | **Change** | | | | **Risk vs. no risk** | |
| --- | --- | --- | --- | --- | --- | --- | --- | --- | --- | --- | --- | --- | --- |
| Outcome | Risk factor | Groups | N | M | SD | M | SD | M | SD | L_CI | U_CI | d | p |
| WHO-5 | COVID-19 related loss ^*^ | Yes | 165 | 71.7 | 20.2 | 61.3 | 25.3 | -10.5 | 20.9 | -15.4 | -5.5 | -0.16 | 0.061 |
| WHO-5 | COVID-19 related loss ^*^ | No | 3,983 | 73.3 | 19.7 | 65.9 | 24.1 | -7.4 | 17.6 | -8.3 | -6.4 |  |  |
| ‘P-score’ | COVID-19 related loss ^*^ | Yes | 165 | 28.2 | 20.5 | 40.0 | 23.4 | 11.8 | 16.8 | 7.0 | 16.5 | 0.15 | 0.084 |
| ‘P-score’ | COVID-19 related loss ^*^ | No | 3,983 | 23.9 | 19.5 | 33.4 | 22.3 | 9.5 | 14.9 | 8.6 | 10.4 |  |  |
| WHO-5 | BMI-group ^2*^ | BMI 30+ | 845 | 73.6 | 20.8 | 65.9 | 25.4 | -7.8 | 17.7 | -10.0 | -5.5 | -0.02 | 0.633 |
| WHO-5 | BMI-group ^2*^ | BMI <30 | 3,303 | 73.1 | 19.4 | 65.7 | 23.8 | -7.4 | 17.8 | -8.5 | -6.4 |  |  |
| ‘P-score’ | BMI-group ^2*^ | BMI 30+ | 845 | 23.3 | 20.4 | 33.0 | 23.0 | 9.7 | 15.9 | 7.6 | 11.8 | 0.01 | 0.803 |
| ‘P-score’ | BMI-group ^2*^ | BMI <30 | 3,303 | 24.3 | 19.3 | 33.8 | 22.2 | 9.5 | 14.8 | 8.5 | 10.5 |  |  |
| WHO-5 | age ^3*^ | >30 yrs | 3,314 | 74.7 | 19.7 | 67.2 | 24.4 | -7.5 | 17.4 | -8.6 | -6.5 | -0.01 | 0.757 |
| WHO-5 | age ^3*^ | <=30 yrs | 834 | 67.2 | 18.5 | 59.9 | 22.2 | -7.3 | 19.3 | -9.3 | -5.4 |  |  |
| ‘P-score’ | age ^3*^ | >30 yrs | 3,314 | 22.2 | 19.3 | 31.7 | 22.4 | 9.5 | 14.9 | 8.5 | 10.5 | -0.03 | 0.439 |
| ‘P-score’ | age ^3*^ | <=30 yrs | 834 | 31.6 | 18.5 | 41.5 | 20.6 | 9.9 | 15.3 | 8.0 | 11.8 |  |  |
| WHO-5 | employed ^4*^ | No | 1,765 | 73.0 | 21.2 | 64.7 | 25.5 | -8.3 | 17.2 | -9.8 | -6.7 | -0.08 | 0.012 |
| WHO-5 | employed ^4*^ | Yes | 2,383 | 73.3 | 18.5 | 66.4 | 23.1 | -6.9 | 18.2 | -8.1 | -5.7 |  |  |
| ‘P-score’ | employed ^4*^ | No | 1,765 | 23.8 | 20.2 | 33.9 | 23.0 | 10.1 | 15.2 | 8.7 | 11.5 | 0.06 | 0.048 |
| ‘P-score’ | employed ^4*^ | Yes | 2,383 | 24.3 | 19.0 | 33.5 | 21.9 | 9.2 | 14.9 | 8.0 | 10.3 |  |  |
| WHO-5 | employment in healthcare ^5*^ | Yes | 268 | 74.3 | 17.9 | 65.3 | 23.2 | -9.0 | 18.9 | -12.6 | -5.5 | -0.13 | 0.051 |
| WHO-5 | employment in healthcare ^5*^ | No | 2,115 | 73.2 | 18.6 | 66.6 | 23.1 | -6.6 | 18.0 | -7.9 | -5.4 |  |  |
| ‘P-score’ | employment in healthcare ^5*^ | Yes | 268 | 27.0 | 20.6 | 36.5 | 22.1 | 9.4 | 15.8 | 5.8 | 13.1 | 0.02 | 0.756 |
| ‘P-score’ | employment in healthcare ^5*^ | No | 2,115 | 23.9 | 18.8 | 33.1 | 21.9 | 9.1 | 14.7 | 7.9 | 10.4 |  |  |
| WHO-5 | COVID-19 positive ^6*^ | Positive | 334 | 70.7 | 18.7 | 60.6 | 23.5 | -10.1 | 18.1 | -13.3 | -6.9 | -0.07 | 0.266 |
| WHO-5 | COVID-19 positive ^6*^ | Negative | 1,592 | 73.2 | 20.1 | 64.3 | 25.0 | -8.9 | 18.7 | -10.5 | -7.3 |  |  |
| ‘P-score’ | COVID-19 positive ^6*^ | Positive | 334 | 26.9 | 19.4 | 38.0 | 20.9 | 11.1 | 14.1 | 8.0 | 14.1 | 0.04 | 0.497 |
| ‘P-score’ | COVID-19 positive ^6*^ | Negative | 1,592 | 24.2 | 19.9 | 34.6 | 23.1 | 10.5 | 15.6 | 9.0 | 12.0 |  |  |
| WHO-5 | gender ^7*^ | Female | 2,181 | 72.4 | 19.5 | 63.4 | 24.4 | -8.9 | 19.3 | -10.2 | -7.6 | -0.17 | <.001 |
| WHO-5 | gender ^7*^ | Male | 1,967 | 74.1 | 19.8 | 68.2 | 23.6 | -5.9 | 15.8 | -7.3 | -4.5 |  |  |
| ‘P-score’ | gender ^7*^ | Female | 2,181 | 25.8 | 19.1 | 36.2 | 22.5 | 10.4 | 15.9 | 9.2 | 11.7 | 0.12 | <.001 |
| ‘P-score’ | gender ^7*^ | Male | 1,967 | 22.2 | 19.8 | 30.8 | 21.9 | 8.6 | 13.9 | 7.3 | 9.9 |  |  |
| WHO-5 | mental disorder ^8*^ | Yes | 703 | 62.0 | 22.8 | 51.5 | 26.0 | -10.6 | 21.4 | -13.1 | -8.0 | -0.19 | <.001 |
| WHO-5 | mental disorder ^8*^ | No | 3,445 | 75.5 | 18.2 | 68.6 | 22.7 | -6.9 | 16.9 | -7.8 | -5.9 |  |  |
| ‘P-score’ | mental disorder ^8*^ | Yes | 703 | 34.6 | 20.1 | 49.2 | 21.5 | 14.6 | 17.1 | 12.4 | 16.8 | 0.39 | <.001 |
| ‘P-score’ | mental disorder ^8*^ | No | 3,445 | 22.0 | 18.7 | 30.5 | 21.2 | 8.5 | 14.3 | 7.6 | 9.5 |  |  |
| WHO-5 | physical disorder ^9*^ | Yes | 2,239 | 72.3 | 21.0 | 64.1 | 25.3 | -8.2 | 17.8 | -9.5 | -6.8 | -0.08 | 0.009 |
| WHO-5 | physical disorder ^9*^ | No | 1,909 | 74.3 | 18.0 | 67.6 | 22.5 | -6.7 | 17.8 | -8.0 | -5.4 |  |  |
| ‘P-score’ | physical disorder ^9*^ | Yes | 2,239 | 23.3 | 19.6 | 34.1 | 22.9 | 10.8 | 15.3 | 9.5 | 12.0 | 0.18 | <.001 |
| ‘P-score’ | physical disorder ^9*^ | No | 1,909 | 25.0 | 19.4 | 33.2 | 21.7 | 8.1 | 14.5 | 6.8 | 9.5 |  |  |
| WHO-5 | immigrant ^10*^ | Yes | 284 | 71.4 | 19.9 | 61.1 | 24.9 | -10.3 | 20.6 | -14.0 | -6.6 | -0.10 | 0.109 |
| WHO-5 | immigrant ^10*^ | No | 2,308 | 73.1 | 19.9 | 64.8 | 24.6 | -8.3 | 18.0 | -9.6 | -7.0 |  |  |
| ‘P-score’ | immigrant ^10*^ | Yes | 284 | 29.6 | 21.2 | 41.1 | 23.9 | 11.5 | 17.4 | 7.8 | 15.2 | 0.08 | 0.242 |
| ‘P-score’ | immigrant ^10*^ | No | 2,308 | 23.3 | 19.5 | 33.6 | 22.7 | 10.2 | 15.0 | 9.0 | 11.5 |  |  |
| WHO-5 | large city ^11*^ | Yes | 957 | 71.7 | 20.7 | 63.3 | 24.5 | -8.4 | 17.7 | -10.4 | -6.4 | -0.07 | 0.071 |
| WHO-5 | large city ^11*^ | No | 3,191 | 73.6 | 19.4 | 66.4 | 24.0 | -7.2 | 17.8 | -8.3 | -6.2 |  |  |
| ‘P-score’ | large city ^11*^ | Yes | 957 | 25.1 | 20.3 | 34.9 | 23.1 | 9.8 | 15.3 | 7.8 | 11.7 | 0.02 | 0.600 |
| ‘P-score’ | large city ^11*^ | No | 3,191 | 23.8 | 19.2 | 33.3 | 22.2 | 9.5 | 14.9 | 8.5 | 10.5 |  |  |

Previously in literature identified eleven risk factors: ^*^ having lost someone due to COVID-19, ^2*^ being obese, BMI 30 or higher, ^3*^ being under age of 30 years old, ^4*^ being unemployed, ^5*^ having healthcare worker employment, ^6*^ having had a COVID-19 infection, ^7*^ being female, ^8*^ having had a mental disorder, ^9*^ having had a physical disorder, ^10*^ having first-generation immigrant status, ^11*^ living in a large city

**Supplementary Figure 2. The percentage of survey respondents rating the importance of each coping strategy as very important and somewhat important during the pandemic.**


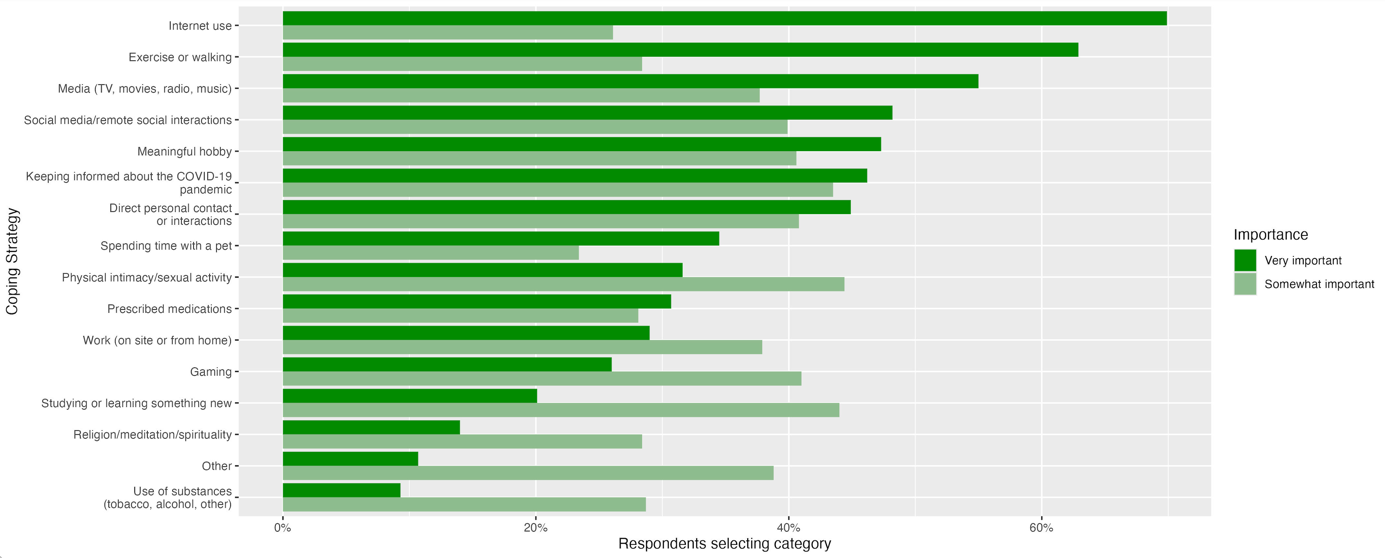

Supplement: Supplementary file 1 [file Supplementaryfile1.docx]
